# A Comparative Loading and Release Study of Vancomycin from a Green Mesoporous Silica

**DOI:** 10.3390/molecules27175589

**Published:** 2022-08-30

**Authors:** Georgiana Dolete, Bogdan Purcăreanu, Dan Eduard Mihaiescu, Denisa Ficai, Ovidiu-Cristian Oprea, Alexandra Cătălina Bîrcă, Cristina Chircov, Bogdan Ștefan Vasile, Gabriel Vasilievici, Anton Ficai, Ecaterina Andronescu

**Affiliations:** 1 Department of Science and Engineering of Oxide Materials and Nanomaterials, Faculty of Chemical Engineering and Biotechnologies, University POLITEHNICA of Bucharest, Gh. Polizu 1-7, 011061 Bucharest, Romania; 2 National Center for Micro and Nanomaterials, University POLITEHNICA of Bucharest, Splaiul Independentei 313, 060042 Bucharest, Romania; 3 BIOTEHNOS SA, Gorunului Street 3-5, 075100 Otopeni, Romania; 4 Department of Organic Chemistry “Costin Nenițescu”, Faculty of Chemical Engineering and Biotechnologies, University POLITEHNICA of Bucharest, Gh. Polizu 1-7, 011061 Bucharest, Romania; 5 Department of Inorganic Chemistry, Physical Chemistry, and Electrochemistry, Faculty of Chemical Engineering and Biotechnologies, University POLITEHNICA of Bucharest, Gh. Polizu 1-7, 011061 Bucharest, Romania; 6 Academy of Romanian Scientists, Ilfov Street 3, 050044 Bucharest, Romania; 7 National Institute for Research & Development in Chemistry and Petrochemistry—ICECHIM, Splaiul Independentei 202, 060021 Bucharest, Romania

**Keywords:** drug delivery, mesoporous silica, wormhole porosity, vacuum-assisted loading, solvent evaporation, physical mixing

## Abstract

Since its first use as a drug delivery system, mesoporous silica has proven to be a surprisingly efficient vehicle due to its porous structure. Unfortunately, most synthesis methods are based on using large amounts of surfactants, which are then removed by solvent extraction or heat treatment, leading to an undesired environmental impact because of the generated by-products. Hence, in the present study, we followed the synthesis of a silica material with a wormhole-like pore arrangement, using two FDA-approved substances as templates, namely Tween-20 and starch. As far as we know, it is the first study using the Tween-20/starch combo as a template for mesoporous silica synthesis. Furthermore, we investigated whether the obtained material using this novel synthesis had any potential in using it as a DDS. The material was further analyzed by XRD, TEM, FT-IR, N_2_ adsorption/desorption, and DLS to investigate its physicochemical features. Vancomycin was selected as the active molecule based on the extensive research engaged towards improving its bioavailability for oral delivery. The drug was loaded onto the material by using three different approaches, assuming its full retention in the final system. Thermal analysis confirmed the successful loading of vancomycin by all means, and pore volume significantly decreased upon loading, especially in the case of the vacuum-assisted method. All methods showed a slower release rate compared to the same amount of the pure drug. Loadings by physical mixing and solvent evaporation released the whole amount of the drug in 140 min, and the material loaded by the vacuum-assisted method released only 68.2% over the same period of time, leading us to conclude that vancomycin was adsorbed deeper inside the pores. The kinetic release of the three systems followed the Higuchi model for the samples loaded by physical mixing and vacuum-assisted procedures, while the solvent evaporation loading method was in compliance with the first-order model.

## 1. Introduction

The possibility of controlling pore sizes and surface chemistry of porous materials makes them extraordinary candidates for the encapsulation of various substances, from small molecules to macromolecules. Consequently, porous materials have demonstrated huge potential in a wide variety of applications, from catalysis to molecular sensing, and even biomedical applications. Metal-organic frameworks (MOFs) are a category of materials that, thanks to their high porosity and tunable composition, have been widely explored in various fields such as catalysis and adsorption [1]. UiO-66, for instance, has improved redox/acidity properties, making it a much better catalyst for breaking down pollutants like toluene and o-dichlorobenzene in the presence of water [2,3]. Also, MOFs have been employed as delivery vehicles for drugs [4] or imaging agents [1,5]. Nevertheless, current limitations of MOFs include, among others, their in vivo metabolic activity [6]. Despite their enormous potential for biomedical engineering, several factors should be considered to ensure adequate biocompatibility. The metal ions used in biological MOFs are limited to elements such as Ca, Mg, Zn, Fe, Ti, or Zr, which are considered essential trace minerals [7]. However, care must be taken considering that the recommended daily doses for these elements are limited to a few micrograms per day. Also, the selection of the appropriate organic ligands must be done carefully. Although the use of ligands that are synthesized in the human body would be indicated, they induce limitations regarding the stability and porosity of the final system [7,8]. Mesoporous silica materials (MSMs) are another class of porous materials that are extensively explored as catalysts [9,10], adsorbents for toxic substances [11,12,13] and, last but not least, have demonstrated outstanding efficiency in designing novel drug delivery systems [14]. By virtue of its well-known biocompatibility and lower costs, compared to MOFs, mesoporous silica is somehow preferred by pharmaceutical industry. Several types of MSMs have been synthesized by modifying the starting precursors and the reaction parameters, leading to a wide variety of materials in terms of pore size and/or geometry, with MCM-41 [15,16] and SBA-15 [17,18] being perhaps the most extensively researched for biological applications. Other well-known mesostructured silicas include MCM-48 with a cubic pore arrangement, MCM-50 with a lamellar layout, or COK-12 with a 2D hexagonal arrangement [19]. Also, materials with unordered pore symmetry and shapes, like TUD-1 [20,21], have been produced, even though their application as drug delivery carriers has not been thoroughly explored. As far as we know, TUD-1 with sponge-like mesopores has been evaluated as a carrier for ibuprofen delivery, being able to release 60% of the drug after 15 min and 96% after 210 min [22,23]. Usually, mesoporous silica synthesis makes use of substantial quantities of cationic surfactants or block copolymer templates, which are further removed to make the structure porous [24]. Unfortunately, template removal involves heat treatment or solvent extraction, and their by-products have a negative impact on the environment. The decomposition of quaternary ammonium salts, for example, generates organic amines [25], which are a severe threat to aquatic life. Moreover, the removal is not complete, and the surfactant residues remain in the material structure. Calcination is the gold standard for the removal of organic templates used in synthesis but has several drawbacks when it comes to large-scale production. The process wastes all of the expensive organic templates and takes a lot of time and energy [26]. Therefore, shorter calcination times or even complete elimination of this step are significant financial-related objectives that the pharmaceutical industry is seeking to achieve. Several other physical methods, such as ultrasounds [24], supercritical fluids (SF) [27,28], plasma technology, ozone treatment [29], or microwave assisted treatment [30], as well as chemical methods, such as ionic liquid treatment [31] or solvent extraction [32], have been tried to get rid of the templates.

Some approaches have been made to overcome the limitations previously described, such as using biocompatible and biodegradable templates. Non-ionic surfactants, which are often used as fillers or emulsifiers in cosmetics, food, or pharmaceutical products [33], have been suggested as a good alternative for synthesizing porous materials. Typically, the majority of non-ionic surfactants have hydrophilic groups attached to poly (ethylene oxide) (PEO) chains [34]. One study showed that a portion of the PEO block lengths or volumes determines the size of mesopores when using oligomeric alkyl poly (ethylene oxide) [35]. In the case of sorbitan ester surfactants, the saturated chain of polysorbate influences the pore diameter. While the laurate chain present in Tween-20 showed capability to produce mesopores with an average pore diameter of 3 nm, the palmitic chain of Tween-40 could reach up to a 5 nm average pore size [36]. In the context of ecologically friendly methods, natural polysaccharides have also been previously exploited as a green template to produce nanostructured materials [37]. They are biodegradable and economical since starch can be extracted from several sources such as vegetables and grains. In addition, it is well known that polysaccharides produce dynamic intramolecular or intermolecular associations, generating coordinative interactions with different transitional metal ions, which play a crucial role in the creation of metal oxide nanoparticles [25]. The chemical structure of starch is composed of linear amylose and branched amylopectin units and possesses very reactive functional groups like hydroxyls (OH) and aldehydes (COH), which promotes the adsorption of different precursors on its surface and facilitates the synthesis of various nanostructured materials, including the self-assembly of silica [38,39].

Due to its potential to combat methicillin-resistant *Staphylococcus aureus*, vancomycin is one of the most commonly used antibiotics for infections. Vancomycin is a glycopeptide antibiotic that binds to D-alanyl-D-alanine subunits and stops peptidoglycan polymerase and transpeptidation reactions. This stops gram-positive bacteria from making their cell walls [40]. Classic route of administration for vancomycin is parenteral, mainly because of its poor membrane permeability and transcellular transport. Besides, its hydrophilic nature leads to a very fast release. Due to economic reasons, efforts have been made to develop systems that could make possible an oral administration of vancomycin [41,42]. Vancomycin entrapment or adsorption into the pores of silica materials have been used for improving its permeability [42]. Several silica-based substrates have been investigated for bonding the drug, due to its highly reactive functional groups that could form hydrogen bonds with the hydroxyl groups present on silica [40]. Qi et al. [43] proved that modification of mesoporous silica with vancomycin also enhanced the efficiency of the drug in targeting and killing gram-positive bacteria. 

This study aims to synthesize a mesoporous silica material with a wormhole-like pore system via a “green” synthesis by replacing expensive and hazardous surfactants like CTAB with Tween-20 and starch, which are FDA-approved. Vancomycin was further loaded for comparative purposes using three different methods: physical mixing, solvent evaporation with a rotary evaporator, and a vacuum-assisted method. 

## 2. Materials and Methods

### 2.1. Materials

Vancomycin hydrochloride, sodium hydroxide, and glacial acetic acid were purchased from Sigma-Aldrich (St. Louis, MO, USA), whereas Tween-20 and sodium silicate were from Santa Cruz Biotechnology, and starch from Merck. Reagents used for preparing SBF were also purchased from Sigma Aldrich (NaCl, NaHCO_3_, KCl, K_2_HPO_4_∙3H_2_O, MgCl_2_∙6H_2_O, HCl, CaCl_2_, Na_2_SO_4_) and Merck ((CH_2_OH)_3_CNH_2_). All reagents were used without further purification. 

### 2.2. Methods

#### 2.2.1. Mesoporous Silica Synthesis

The mesoporous silica material was obtained using sodium silicate as the silica precursor and a dual templating method by combining Tween-20 as a directing agent and starch as a co-template. Briefly, an alkaline solution was prepared by dissolving 4.2 g of NaOH in 135 mL of ultrapure water. A quantity of 20 g of sodium trisilicate was gradually added to the alkaline solution, assisting its dissolution by magnetic stirring at 250 °C. The solution obtained was cooled to room temperature under a stream of cold water. The surfactant solution was prepared separately by mixing 1 mL of Tween-20 with 45 mL of ultrapure water. After homogenization, 0.5 g of starch was slowly added, and we waited for its complete dissolution. The cooled alkaline solution was added to the surfactant under magnetic stirring. Finally, the last suspension was precipitated with a solution of acetic acid 7.5% (*v*/*v*) using ammonium hydroxide as a catalyst. The precipitate was washed several times with ultrapure water (18.2 MΩ). Lastly, the template was cast away via a triple extraction method using a total amount of 600 mL of ethanol, filtered, and dried in a vacuum oven at 50 °C for 12 h. The dried powder was further characterized and used as a support for loading the antibiotic in three different ways. 

#### 2.2.2. Drug Loading Process

Given the fact that one of our objectives was to compare release kinetics for different loading procedures, we selected three widely used methods, one of which is a solvent-free method. Each method aimed at loading the material with 30% vancomycin (*w*/*w*) to the total mass of the drug delivery system. 

Physical mixing involved a light blending of the bulk MSM powder (in a ratio of 70:30 *w*/*w*) with the required amount of vancomycin, and the material was labeled as MSM@Van_PM. 

Solvent evaporation adsorption consisted of dispersing the MSM in a vancomycin solution containing the amount of drug required to obtain an MSM: Van weight ratio of 70:30 after solvent removal. The dispersion step was performed in an ultrasonic bath for 20 min at room temperature, and the solvent was further removed by using a rotary evaporator at 40 °C, 0.1 bar pressure. The obtained powder was further dried in an oven for 12 h to allow complete evaporation of any residual solvent and tagged as MSM@Van_SE. 

Finally, the third method, namely vacuum-assisted loading, consisted of the solubilization of 90 mg of vancomycin in 2 mL of water. The method is presented in more detail in our previous work [16]. Once the vacuum was removed, the drug solution that reached the pores is adsorbed deeper into the pores. Subsequently, the material was also dried in the oven so that all the solvent could evaporate, and was identified as MSM@Van_VA.

#### 2.2.3. Physico-Chemical Characterization of the Materials

Both low angle and high angle (XRD) X-ray diffraction patterns were carried out using a PAN Analytical Empyrean powder diffractometer (PANalytical, Almelo, The Netherlands) equipped with CuKα radiation (λ = 1.541874 Å). Diffractograms were assessed within 2θ = 0.5–10, and 2θ = 10–80, respectively.

The materials were further characterized by infrared spectroscopy to identify the structural changes following the loading step. FT-IR spectra were collected using a Nicolet iS50 FTIR equipped with a DTGS ATR detector, by co-addition of 64 scans in the range 4000–400 cm^−1^, at resolution 8, collecting background before every sample. 

Textural features of the materials were determined by N_2_ physisorption measurements at 77 K, using a NOVA 2200e-Quantachrome Analyzer porosimeter. The specific surface area was calculated from the adsorption isotherm using the Brunauer–Emmett-Teller equation. Total pore volume was estimated from the amount of N_2_ adsorbed at the relative pressure P/P_0_ = 0.9 and pore size distribution was obtained from the isotherm desorption applying the Barett–Joyner–Halenda method. 

Loading efficiencies for each method were calculated using data obtained from thermogravimetric analysis combined with differential scanning calorimetry (TGA-DSC). The size of the unloaded and loaded material was investigated using dynamic light scattering (DLS). Using the ultrasonic bath, the samples were dispersed homogenously in ethanol at a concentration of 0.35 mg/mL and a small amount was placed inside the measurement cell of the DelsaMax Pro equipment (Backman Coulter, Brea, CA, USA). For each sample, measurements were performed in triplicate.

#### 2.2.4. Drug Release and Dissolution Kinetic

Vancomycin delivery studies were conducted in an SBF solution prepared by the Kokubo protocol [44]. Approximately 50 mg of each drug-loaded material was weighed in filter paper bags, immersed in 100 mL of SBF, and kept under continuous stirring at 37 °C. The release profiles were achieved in real-time using a peristaltic pump driving the solution to a quartz flow cell over 3 h. The amount of drug released was calculated based on the Lambert–Beer equation. 

Table 1 presents the mathematical models that were used to study the release kinetics of the three vancomycin-loaded systems. The best fit model for each individual sample was determined by analyzing the regression coefficient values (R^2^), after plotting the graphs as specified in the table. 

The zero-order kinetic model was chosen due to the fact that an ideal drug delivery system should follow this model, which assumes that the therapeutic substance is released at a constant rate for a period of time, independent of concentration. Also, devices that follow this model are supposably releasing the drug at the same rate that is cleared from the body, which enables stable drug plasma concentrations without need for redosing the therapeutic substance [45]. The other two models were selected based on the composition of the final release systems as follows: the release of active substances from porous matrices, including those based on mesoporous silica materials, usually show compliance with the Higuchi model, while hydrophilic drugs often follow the first-order model [46].

**Table 1:** Release kinetics models applied [47].

Release Kinetic	Equation	Plot Representation
Zero-order	$C_{t}=C_{0}+K_{0}\cdot t$	cumulative % drug released vs. time
First order	${\log\;C}_{t}={\log\;C}_{0}-K_{1}\cdot \frac{t}{2.303}$	log cumulative %drug remaining vs. time
Higuchi model	$C_{t}=K_{H}\cdot \;\sqrt{t}$	cumulative % drug released vs. square root of time

C_t_—drug released in time ‘t’; C_0_—the initial amount of drug in dissolution medium (usually is 0); K_0_—zero order release constant; K_1_—first order release constant; K_H_—Higuchi dissolution constant.

## 3. Results and Discussions

The obtained mesoporous silica material (MSM) was characterized by different analytical methods. XRD and TEM analyses were performed only on the synthesized material, while FTIR spectroscopy, N_2_ adsorption/desorption, dynamic light scattering and thermal analysis were employed both for the unloaded and loaded materials, to confirm and quantify the presence of vancomycin in the final release systems. The release studies were accomplished only on the loaded materials.

**Figure 1:**
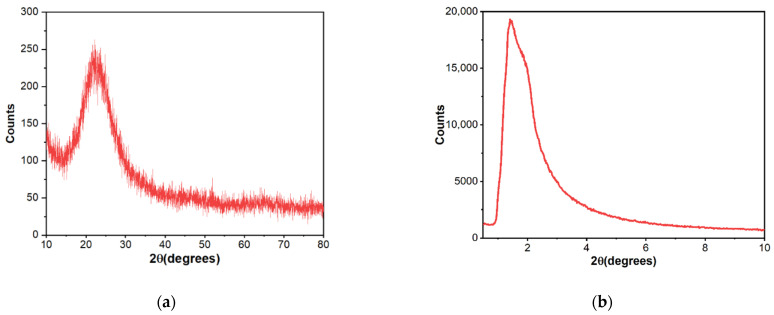
High-angle (**a**) and low-angle (**b**) X-ray diffraction patterns of mesoporous silica material.

**Figure 2:**
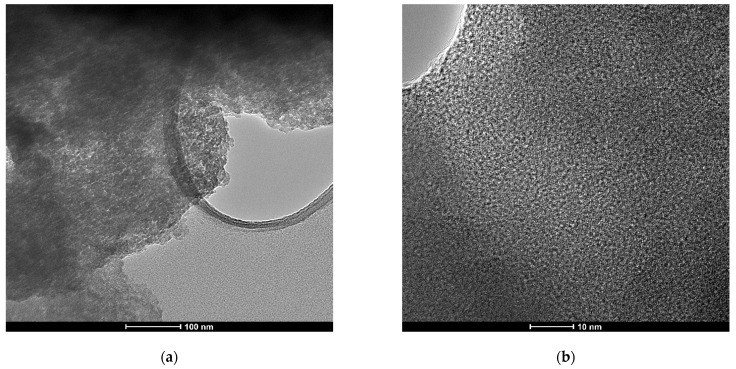
TEM images of as-synthesized mesoporous silica at different magnifications: (**a**) scale-bar 100 nm and (**b**) scale-bar 10 nm.

As Figure 1 shows, X-ray diffraction exhibited patterns typical for mesoporous silica. The broad hump at 2θ = 27.13° obtained at high-angle powder XRD is specific to amorphous silica. On top of that, the low-angle XRD pattern depicts a strong, broad peak with a higher angle shoulder at approximately 2θ = 1.5°, showing a linked distribution of framework pores but no regular pore structure [48,49,50]. The data is consistent with other studies where non-ionic surfactants have been used as templates [51]. The amorphous, porous nature was substantiated by TEM images (Figure 2), where it is visible that the material presents a disordered pore distribution, typical for wormhole-like arrangement [50,52]. The wormhole-like channels are randomly, still homogeneously distributed throughout the bulk phase. 

**Figure 3:**
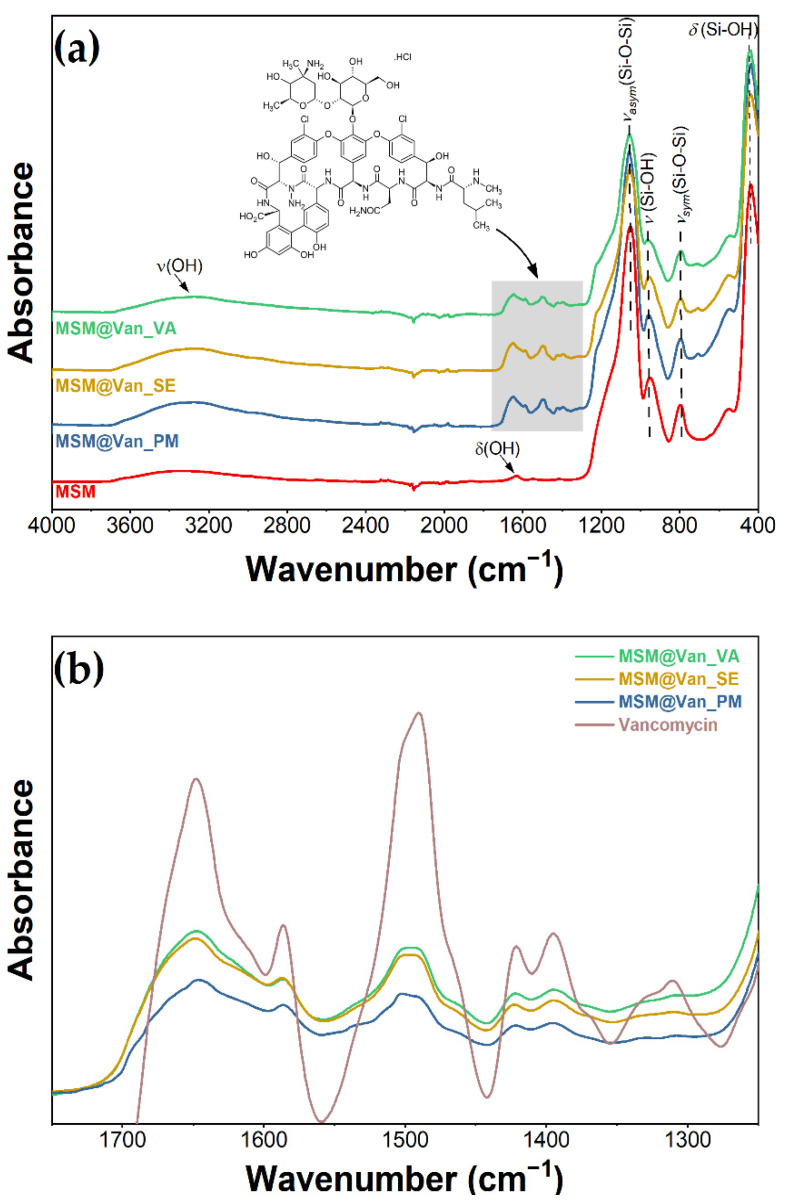
Infrared spectra (**a**) before and after loading with vancomycin; (**b**) overlapped spectra of loaded materials and vancomycin.

As-synthesized MSM and loaded MSM were investigated by infrared spectroscopy for the detection of present functional groups (Figure 3). FT-IR data showed the presence of bands of absorption typical for mesoporous silica. Stretching and bending vibrations of surface hydroxyl groups are put into evidence by the existence of a broad peak between 3600 and 3200 cm^−1^, as well as a small peak appearing at 1631 cm^−1^ [53]. At approximately 1050 cm^−1^, we can see the sharp peak that corresponds to asymmetric stretching vibrations of Si-O-Si, complementing the adsorption band at 795 cm^−1^, which is characteristic of symmetric stretching vibrations belonging to the same siloxane group [54]. At 958 and 438 cm^−1^, we can see the peaks associated with rocking and stretching vibrations that occur in the Si-O bond of the silanol functional group [55]. Notably, there is an absence of absorption bands characteristic of starch or Tween-20, thus indicating their efficient removal by the ethanol extraction method. When the materials were loaded using the three different methods, the spectra visibly changed when compared to the bulk material. The highlighted area from Figure 3a corresponds to absorption bands characteristic of vancomycin: 1649 cm^−1^ corresponds to C=O stretching vibrations; 1498 cm^−1^ appears due to stretching vibrations of the C=C, and the peak at 1394 cm^−1^ relates to C-C bending vibrations [56]. Additionally, for vancomycin-loaded MSM, a variation in the intensity of the absorption bands occurred, compared to bare MSM. The peaks corresponding to Si-O-Si (1050 cm^−1^ and 795 cm^−1^) and Si-OH (958 cm^−1^ and 438 cm^−1^) decreased in intensity when loaded with vancomycin, because of intermolecular interactions between the drug and the support. Moreover, by zooming in the area between 1700 and 1350 cm^−1^, overlapping the spectra at a matched scale (Figure 3b), and comparing the pure vancomycin with our materials, we confirmed that the region corresponds to the drug. Moreover, the intensity of the 958 cm^−1^ adsorption peaks changes differently, depending on the loading method. This decrease denotes that interactions between vancomycin and MSM are differently achieved for all loaded samples. The peak has the lowest intensity for the MSM@Van_VA, followed by MSM@Van_SE and eventually by MSM@Van_PM. It is worth mentioning that MSM@Van_PM has the intensity comparable to pristine MSM, evidence that most of the drug is probably found as independent phase in the mixture. 

**Figure 4:**
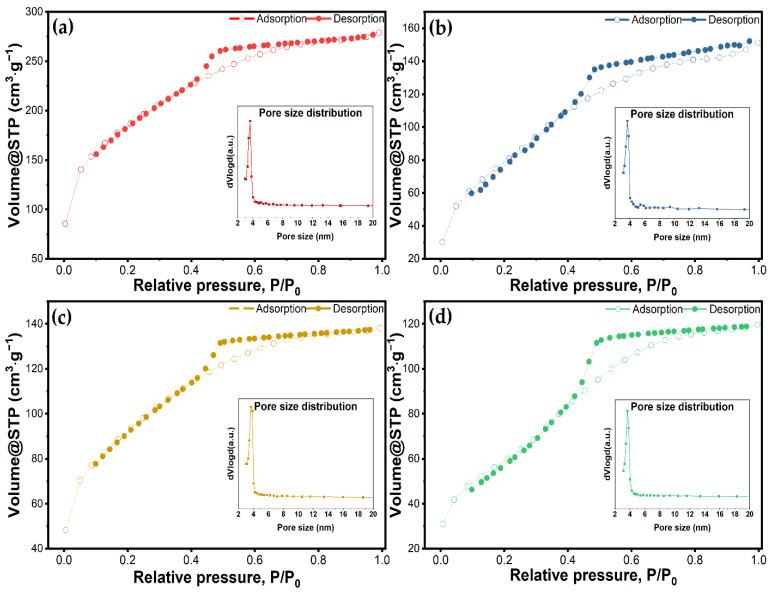
N_2_ adsorption/desorption isotherms and corresponding pore size distributions for (**a**) unloaded MSM, and loaded MSMs by (**b**) physical mixing, (**c**) solvent evaporation, and (**d**) vacuum-assisted method.

Textural features such as surface area, pore volume, and pore size distribution of nanoparticles have a key role in drug loading capacity, drug dissolution, and drug release kinetics. Figure 4 depicts the nitrogen adsorption/desorption isotherms of the bulk material and the vancomycin-loaded materials. Data regarding BET surface area and pore volume are given in Table 2**.** All the samples showed a type IV isotherm, with an H_2_ hysteresis characteristic of materials with a disordered distribution of size and shape according to the IUPAC classification [57]. Other studies using Tween-20 as a directing agent have also demonstrated this characteristic isotherm [36]. The adsorption curves exhibit a steep rise at a relative pressure lower than 0.1, which indicates the monolayer has been completed and multilayer adsorption begins. All the materials maintained the same trends for their isotherms after vancomycin had been loaded, but the volume of adsorbed N_2_ was significantly reduced. The as-synthesized material had a BET specific surface area and a mesopore volume of 642 m^2^∙g^−1^ and 0.4313 cm^3^∙g^−1^, respectively. Following the loading processes, the surface area for all three materials was reduced 1.96, 1.99, and 2.94 times for MSM@Van_PM, MSM@Van_SE, and MSM@Van_VA. This reduction in the surface area suggests that the antibiotic has been successfully loaded either on the surface or in the pores of the material. Nevertheless, the vacuum-assisted loading methodology showed a significantly higher decrease in pore volume than the other methods. This may show that a larger amount of vancomycin was adsorbed into the pores and certainly can interact with the inner surface in the case of the vacuum-assisted method. Pore size distributions obtained by applying the BJH method to the adsorption branch of the isotherm showed a maximum at a pore diameter of about 3 nm, which is consistent with other studies where Tween-20 was used [36].

**Figure 5:**
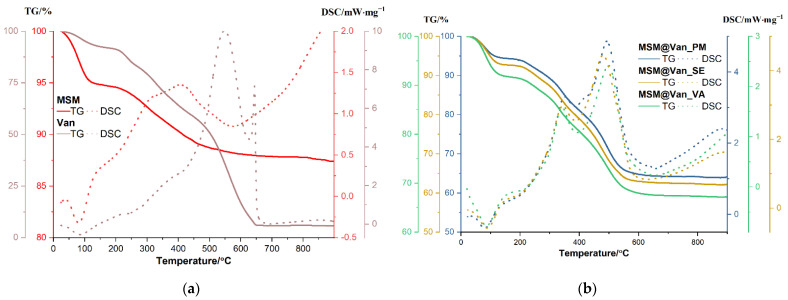
TGA curves and DSC profiles of (**a**) bare mesoporous silica material and vancomycin; (**b**) loaded mesoporous silica materials using the three different loading methods.

Thermal analysis was assessed for the unloaded support and the pure antibiotic, as well as for the three loaded samples (Figure 5). The amount of adsorbed water molecules and the density of silanol groups on the nanoparticles surface can be evaluated by TGA. Up to 150 °C, the sample will lose physically adsorbed water from surface and from pores, that are usually interacting by hydrogen bonds. Condensation of the silanol groups takes place in the interval 150–900 °C, when a continuous, slow mass loss is recorded. This condensation process, accompanied by elimination of water molecules, is responsible for the formation of silica and densification of the structure. The amount of H_2_O and density of silanol groups were calculated as indicated in [16] and are summarized in Table 3. The calculated concentration of silanol groups is quite high, better than the reported values from [16], and similar to those for raw MCM-41 reported in [58].

The unloaded MSM sample is losing 5.16% of initial mass up to 150 °C, the process being accompanied by an endothermic effect with a minimum at 81.3 °C. Most probably, this represents the elimination of water molecules physically adsorbed on the surface of the nanoparticles [16]. Between 150–600 °C, the sample is constantly losing mass, with the recorded value being 6.73%. The corresponding effect is exothermic, broad, and asymmetric, with a maximum at 311.9 °C and one at 407 °C. The process is probably oxidation of organic residuals (Tween/starch used in the synthesis and which, even present in a low amount is not perceptible in FTIR because of low molar absorption of these molecules) from the synthesis used to obtain the material. The residual mass is 87.42%. Pure vancomycin is losing 7.43% of its initial mass between room temperature and 150 °C, and the corresponding effect is endothermic, with a minimum at 85.8 °C. This can be attributed to the removal of water present in the sample. Between 150 and 270 °C, 9.66% of the mass was lost, and another 24.35% was lost between 270 and 450 °C. Both processes are accompanied by weak exothermic effects. The sample undergoes significant oxidative degradation between 450 and 650 °C, with vancomycin losing 52.68% of its mass. The oxidation of the sample is accompanied in this interval by a strong, broad exothermic effect with a maximum of 584.4 °C. The sharp, strong exothermic peak at 644.4 °C is thought to be caused by carbonaceous mass. The residual mass is 5.76%.

Similarly, an initial weight loss, attributed to physisorbed water molecules, is observed for each drug-loaded MSM. Precisely, MSM@Van_PM, MSM@Van_SE, and MSM@Van_VA lose 5.61%, 7.27%, and 8.14% of their initial masses [59]. These weight losses occur between room temperature and 150 °C and are accompanied by endothermic effects with minimum peaks between 80.7–87.8 °C. Compared to the solvent-free loading method, the samples obtained by solvent evaporation and vacuum assisted have a slightly increased number of water molecules, which indicates that solvent residues remain in the samples. Up to 600 °C, the vancomycin-loaded samples follow the same pattern and are constantly losing mass, the recorded values being 29.62% for MSM@Van_PM, 29.76% for MSM@Van_SE, and 23.88% for MSM@Van_VA. The corresponding effects are exothermic and partially overlapped, with maximums that can be found in Table 4.

The process is probably explained by oxidation of the organics present in the sample and the condensation of various silanol groups, followed by water elimination and densification. 

Although all the three loading methods used within the study apparently show a 100% loading efficiency for vancomycin in the final DDSs, data obtained from TGA analysis were exploited for calculating the drug concentration in each of the three drug-loaded materials. This step was necessary so that further predictions regarding the release study are as precise and accurate as possible. The highest amount of vancomycin is found in the material loaded by solvent evaporation, followed by the physical mixing sample, and lately by the vacuum-assisted method (Table 4). These results can be explained considering the working conditions. For instance, in the case of vacuum assisted method, during the addition of the solubilized drug, some solution can be adsorbed by the vacuum pump, and some of the added drug solution is dried onto the glass surface without reaching the mesoporous material (the drying being much faster compared to normal pressure drying).

**Figure 6:**
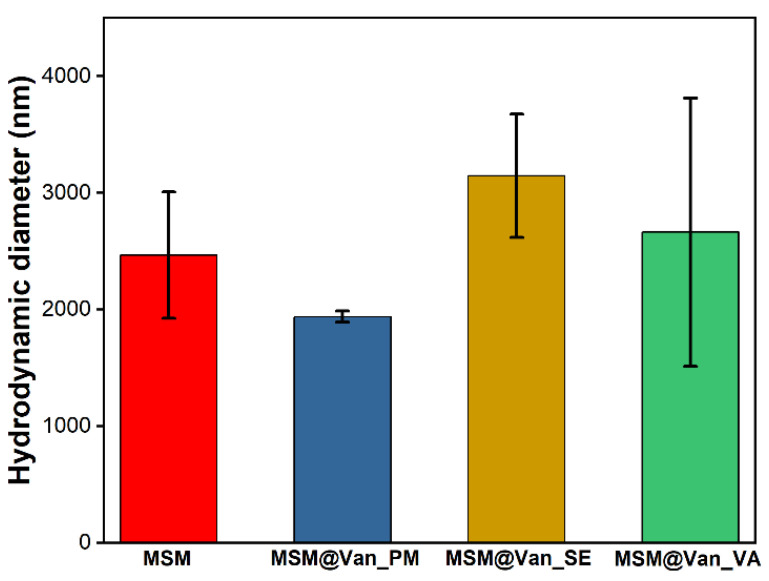
Average hydrodynamic diameter for pristine and vancomycin-loaded MSM.

The hydrodynamic diameter for both the pristine and the vancomycin-loaded samples are given in Figure 6. As it can be seen, the loading method influences these values. Pristine MSM had an average hydrodynamic dimeter of 2465 ± 543 nm. While the samples loaded by solvent-based methods increased their size, MSM@Van_PM suffered a slight decrease. This effect may occur due to the fine grinding process by which the sample was loaded. Comparing the samples obtained by solvent-based methods, the average size of MSM@Van_SE increased 1.27 times, while that of MSM@Van_VA increased 1.07 times. The differences in the growth of particles after loading can be explained based on the fact that, in the case of vacuum-assisted sample, a larger amount of drug was adsorbed into the pores, while the MSM@Van_SE sample most probably presents a higher amount of vancomycin on its surface. 

**Figure 7:**
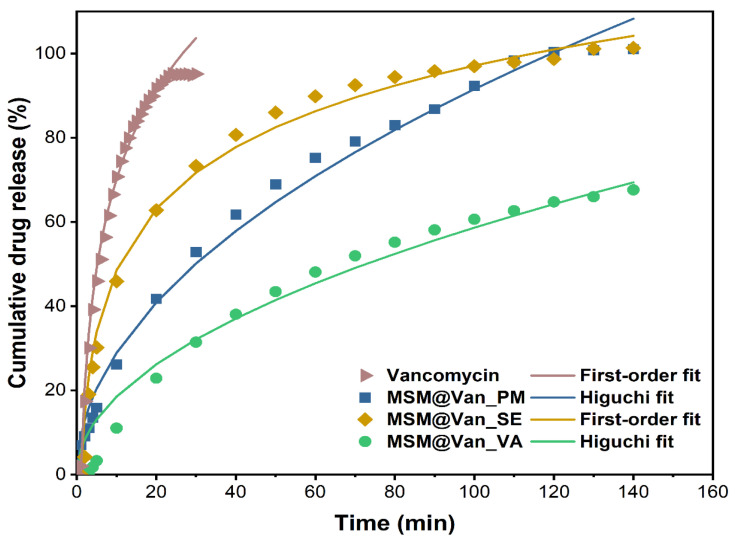
Cumulative drug release percentage of pure vancomycin hydrochloride and vancomycin-loaded mesoporous silica material by different methods. Experimental data are represented by symbols and fitted data are shown by lines.

Regardless of the method used for loading, vancomycin had an extended-release compared to the pure drug. For comparison, the expected maximum amount of vancomycin (15 mg) was also placed in a filter bag, and we waited for its complete dissolution in SBF (until reaching plateau). As Figure 7 depicts, pure vancomycin showed a fast release rate, as expected, reaching its maximum concentration in just 30 min, of which 45.9% was released in the first 5 min. Similar, Ndayishimiye et al. [42] achieved a 82% vancomycin released in PBS buffer after 40 min. Compared to the release of the pure drug, loaded samples showed a slower release. Precisely, in the first 5 min, 13.8%, 25.8%, and 2.0% of vancomycin was released from MSM@Van_PM, MSM_Van@SE, and MSM@Van_VA. The initial high percent cumulative release of vancomycin, for MSM@Van_PM and MSM@Van_SE samples, perhaps because of vancomycin presence on the surface of the MSM. The samples loaded by physical mixing and by solvent evaporation released all the available vancomycin in 140 min. On the other hand, the sample loaded by vacuum assistance released only 68.2% of the total amount of available drug in the respective timeline. Supplementary readings after 24 h and 48 h revealed that MSM@Van_VA continued to release the drug up to 87.72% after 24 h, and eventually released all the available drug (99.18%) somewhere between 24–48 h. Most probably, the vacuum-assisted loading method permitted more vancomycin to be adsorbed in the pores of the material, leading to a more prolonged release in SBF. Doadrio et al. [60] obtained a similar release pattern for unfunctionalized SBA-15 concluding that a weak electrostatic interaction occurs between the positively charged head of vancomycin and the negative surface charge of SBA-15. On the other hand, octadecyl-modified SBA-15 slowed down the vancomycin delivery, but only about 10% vancomycin was released in 2 days. Consequently, the release behavior of the obtained DDSs can be further improved by several approaches, such as surface functionalization or sealing the pores. In any case, care must be taken for surface functionalization since different interactions between the drug and mesostructured material can occur. As demonstrated by Kurczewska et al. [61], weak ionic interactions give a fast release, while strong ionic bonds give a very slow release which after all may be ineffective. The same paper showed the effect of a prolonged release, when vancomycin was covalently attached to the matrix. Anyway, the overall percent release was around 60% in more than a week, which is unsatisfactory for an oral delivery system. Also, composite scaffolds based on gelatin and vancomycin-loaded mesoporous silica for local infected bone defects indicated a prolonged release of vancomycin, up to 30 days [62]. 

**Figure 8:**
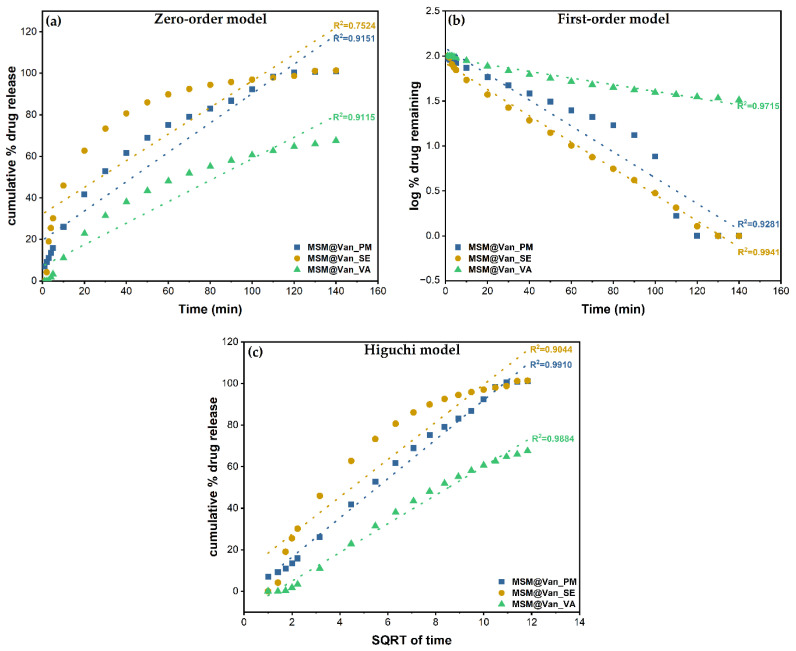
Zero order (**a**), First order (**b**), and Higuchi (**c**) models applied to the three different loading procedures.

The three standard mathematical models, as mentioned in Table 1, have been applied to the obtained experimental data. Correlation coefficients were determined to evaluate the best fitting model for each drug loading procedure. The obtained plots and calculated R^2^ are embodied in Figure 8. The zero-order model showed wick-fitting results for all three samples, with R^2^ equal to 0.9151, 0.7524, and 0.9115 for MSM@Van_PM, MSM@Van_SE, and MSM@Van_VA respectively. In opposition, the first-order model showed good correlation coefficients for MSM@Van_SE (R^2^ = 0.9941) and MSM@Van_VA (R^2^ = 0.9715), with the regression lines going through almost all experimental points in both cases. First-order release usually is representative for water-soluble drugs released in a concentration-dependent manner from a porous matrix and states that the amount of released drug tends to decrease in time [47]. However, by applying the Higuchi model (Figure 8c), the vacuum-assisted loaded sample improved the R^2^ value up to 0.9884 and, consequently, Higuchi was selected as the best fitting model for MSM@Van_VA. MSM@Van_PM also demonstrated a correlation coefficient closer to 1 (R^2^ = 0.9910) after applying the Higuchi equation. Prior studies showed that Higuchi fitting is a common model to describe release of a wide range of molecules from mesoporous silicas, weather of hydrophilic or hydrophobic nature [63]. The release mechanism is similar to that obtained by [60,64], which gives us the premises that this mesoporous silica with wormhole-like porosity would work same as SBA-15 or MCM-41 materials. Based on the Higuchi model, in MSM@Van_PM and MSM@Van_VA samples, vancomycin release is governed by Fickian diffusion. On contrary, the first-order kinetic model fit of the MSM@Van_SE sample indicates a mechanism based on dissolution. The similarity between the sample loaded by solvent evaporation, assisted by rotary evaporator, and the pure drug can be explained through the processes that take place during loading. Precisely, during immersion of bulk MSM in the vancomycin solution, part of the antibiotic solution is absorbed into the pores, while another part is not. When applying the rotavap treatment, the evaporation process takes place in the opposite direction. Thus, in the first phase, the solvent outside the pores evaporates, which leads to a crystallization of vancomycin on the surface of the support, and in the second phase, the solvent evaporates from the pores, keeping the drug inside. The study on the release mechanism reinforces the assumption that a large part of vancomycin is found outside the pores, as indicated by the particle measurements using DLS. The direct effect of the presence of vancomycin on the surface is the release that occurs as a simple dissolution of the drug in the medium, thus the similarity with the pure drug release. 

The first-order constants (K_1_) and Higuchi dissolution constants (K_H_) are derived from the slopes of the plot and given in Table 5. The Higuchi model presented release rate values (K_H_) higher for MSM@Van_PM than for MSM@Van_VA. As a result, vancomycin tends to release more easily achieving a complete release in 140 min. Anyway, both samples exhibited lower values compared to the non-functionalized SBA-15 loaded with vancomycin in [60]. Even though MSM@Van_SE followed the same dissolution mechanism as pure vancomycin, the release rate was approximately 34 time slower compared to pure drug. This is probably due to the interactions that occur between the mesostructured support and drug, but also due to the fact that a small amount of drug was absorbed into the pores. 

## 4. Conclusions

The study aimed to obtain a drug delivery system for vancomycin by using silica with a wormhole-like mesoporous structure. The novelty of the study consists of using Tween-20, a non-ionic surfactant, with starch as a co-template, and by proposing a vacuum-assisted methodology of loading vancomycin within mesoporous silica. X-ray diffractograms and transmission electron micrographs confirmed the formation of the mesoporous silica with wormhole-like pores. Furthermore, N_2_ sorption/desorption analysis of the synthesized material shows the formation of a structure with a large specific area and a large pore volume. Also, pore size distribution reveals a maximum of around 3 nm, thus classifying the materials in the category of mesoporous silica. As the relative total weight loss was around 12.5%, as obtained by thermal analysis of the as-synthesized material, we assume that there was not much high organic matter in the system, so the ethanol extraction efficiently removed the template. All these data indicated that silica with the desired porous arrangement was achieved by using Tween-20 and starch as templates, followed by triple extraction of the template with ethanol. By loading the support with vancomycin, textural properties such as specific area and pore volume were considerably modified. Pore loading proved to be more efficient in the case of the vacuum-assisted method, as confirmed by both the decrease in pore volume and the SBF release studies. During the three-hour test period, 68.2% of the vancomycin was released into SBF. The other two methods, on the other hand, released the full amount of vancomycin. The release kinetics of physical mixing and vacuum-assisted loading methods displayed good compliance with the Higuchi model. The release constant was slower for MSM@Van_VA, and was attributed to the presence of vancomycin deeper in the pores of the material. The sample loaded via solvent evaporation method indicated compliance with the first-order model, like the pure drug. However, the release constant was significantly improved since the total amount of vancomycin was released over a longer period of time. 

## Figures and Tables

**Table 2:** Textural characteristics of unloaded MSM and vancomycin loaded MSMs according to physisorption curves.

Sample Name	Surface Area (m^2^∙g^−1^)	Pore Volume (cm^3^∙g^−1^)	Pore Diameter (nm)
MSM	642.8	0.4313	3.66
MSM@Van_PM	327.6	0.2648	3.66
MSM@Van_SE	322.1	0.2136	3.65
MSM@Van_VA	218.3	0.1850	3.46

**Table 3:** MSM characteristics from TGA values.

Sample	Mass Loss (%)			n_H2O_	n_OH_	N_H2O_	N_OH_
	RT-150 °C	150–900 °C	Residual Mass	(mmol/g)		(Groups/nm^2^)	
MSM	5.16	7.41	87.42	2.87	8.23	1.46	4.21

**Table 4:** Mass loss and loading efficiencies calculated according to TG-DSC curves.

Sample	Mass Loss (%)			Thermal Effects (°C)		Calculated Vancomycin (%)
	20–150 °C	150–600 °C	Residual Mass	Endothermic	Exothermic	
MSM	5.16	6.73	87.42	81.3	311.9 407.0	-
VAN	7.43	86.69	5.76	85.8	584.4 644.4	-
MSM@Van_PM	5.61	29.62	64.06	80.7	350.6 492.1	28.61
MSM@Van_SE	7.27	29.76	62.18	87.8	344.1 479.6	30.91
MSM@Van_VA	8.28	23.88	67.16	85.9	341.3 498.0	24.81

**Table 5:** Correlation coefficients and release constants derived from regression analysis.

	Unit	Van	MSM@Van_PM	MSM@Van_SE	MSM@Van_VA
First order	- min^−1^	R^2^ = 0.9984 K_1_ = 0.1246	R^2^ = 0.9281 K_1_ = 0.0085	R^2^ = 0.9941 K_1_ = 0.0337	R^2^ = 0.9715 K_1_ = 0.0331
Higuchi model	- min^−1/2^	R^2^ = 0.9735 K_H_ = 0.0381	R^2^ = 0.9910 K_H_ = 0.1430	R^2^ = 0.9044 K_H_ = 0.1004	R^2^ = 0.9884 K_H_ = 0.1050

## Data Availability

Available on demand.

## Sample Availability

Samples of the compounds are not available from the authors.
